# History of a Case of Spinal Disease

**Published:** 1849

**Authors:** M. F. Irwin

**Affiliations:** Crystal Lake, Ill.


### ARTICLE VI.

*History of a case of Spinal Disease.* By Dr. M. F. Irwin, of Crystal Lake, Ill.

H. P., aged 23, a farmer, of robust constitution and temperate habits, applied to me, Sept., 20th, 1847, on account of slight pain in the right side and soreness in the abdomen.

Appetite good, bowels regular, tongue and countenance showed no indication of disease. I refused to prescribe medicine, telling him that he had labored too hard, and that he needed little else than diet and temporary rest.

I heard nothing more from this patient until January 2, 1848, when, upon being summoned by his father to see him. He stated to me that since August he had felt slight pain in one or both sides; that at times he had had slight soreness in the abdomen and a shortness of breath, but had labored hard until a week since, when he had given up work, "because" as he expressed himself, " one of his legs refused to go off." Upon examination it was found that his appetite was unimpaired, no unusual tenderness could be detected in the chest or abdomen, or on the track of the spinal column, yet he felt a soreness in the abdomen and lameness in the chest and upper part of the body, upon retching or making a strong effort with the anus. He was unable to move his left leg without the aid of the hands; the right was also partially paralyzed. Sensation, too, was so much impaired in both inferior extremities that deep gashes could be cut in them without his having any knowledge of it.

*Treatment*,—He was bled to the extent of sixteen ounces, and a full dose of submur. hyd. administered, followed by Sulph. sodæ, until free purging followed; diet restricted, and a large blister applied over the region of the spiual column. He was then directed to take mass hyd. six grain, at night, and ten grains pulv. Doverii, occasionally, and to apply sinapisms to the legs and feet.

Saw him again January 8th. Symptoms of paralysis no better, sensation less; he could now be cut upon the lumber region to the depth of half an inch, without experiencing any pain.

The soreness had now entirely left the chest and abdomen, appetite good, pulse regular. There was no loss of sensation or the power of motion, in the arms and upper portions of the body, but in the lower extremities there was complete loss of motion, and there was but little sensation.

It is not necessary to describe the treatment of this case in detail. Suffice it to say that calomel, cupping, scarifying blistering, leeching, sinapisms, iodine, strychnine, and the cold douche were successively or in conjunction tried, but in spite of the combined efforts of the best council at hand, he sunk under the disease April 13th following the January of the attack.

*Autopsy twenty hours after Death.*—The spinal column and spinal cord, were the only parts examined. The examination was as minute as the case would admit. In the cervical region no unusual appearance was detected. The dorsal portion of the cord, on examination, was found to be slightly atrophied opposite the three last dorsal vertebræ. In the the lumber region very different appearances presented themselves. All that could be detected of the spinal marrow at the lower part of this region, was two shre(ʼs or cords about the size of a common knitting kneedle, of a whitish appearance, very tender and easily broken. Still higher up, these cords were found to be more numerous, and seemed each to be constituted of numerous fibres, altogether constituting a complete net-work of cords. Mingled with these fibres, a whitish substance resembling the medullary portions of fibrin might be observed, having the appearance of soft portions of the spinal marrow that had not been absorbed; here, also, might be observed, running along the fibres, innumerable small blood-vessels, almost too minute for the naked eye to detect, yet giving the parts a slight vascular appearance. Still higher, opposite to the last dorsal vertebræ, the cord appeared as if a section of it had been made from behind forward and downward in such a manner as to divide all but the small shreds or cords above described.

The membranes of the cord seemed entire, presenting a very vascular appearance. It may here be observed that the cavity below the last dorsal vertebra and the sacrum was filled with a fluid resembling the serum of the blood.

*Remarks.*—This case of spinal affection presented the following to me unusual phenomena. But little, if any unusual tenderness could at any time be detected along the course of the spinal cord; all pain throughout the course of the disease was referred to the abdomen and chest; notwithstanding before death the integument and muscles sloughed from the sacrum, yet there was generally regularity of the bowels and in the discharge of urine, though fæces and urine occasionally passed involuntarily and without the patient's knowledge.

It seems difficult to account for the origin of such a disease in a young man of so unusually good constitution and discreet and temperate habits. No cause could be assigned by him for the disease, or time fixed when it commenced; our remedies, too, seemed to do him little if any good, the case going on as though nothing had been done.

